# Overcoming intra-tumoral heterogeneity for biomarker discovery in the high-grade serous ovarian cancer proteome

**DOI:** 10.1038/s41698-025-00911-y

**Published:** 2025-06-11

**Authors:** Srikanth S. Manda, Maiken M. Espersen, Cristina Mapagu, Natalie Bouantoun, Jessica Boros, Yoke-Eng Chiew, Sivatharsny Srirangan, Swetansu Pattnaik, Catherine J. Kennedy, Alison H. Brand, Dale W. Garsed, Ahwan Pandey, David D. L. Bowtell, The Australian Ovarian Cancer Study (AOCS) Group, Natasha Lucas, Dylan Xavier, Sadia Mahboob, Daniel Bucio-Noble, Brett Tully, Peter G. Hains, Phillip J. Robinson, Qing Zhong, Roger Reddel, Anna DeFazio, Rosemary L. Balleine

**Affiliations:** 1 ProCan®, Children’s Medical Research Institute, Faculty of Medicine and Health, The University of Sydney, Westmead, NSW Australia; 2 Centre for Cancer Research, The Westmead Institute for Medical Research, The University of Sydney, Westmead, NSW Australia; 3 Faculty of Medicine and Health, The University of Sydney, Sydney, NSW Australia; 4 Department of Medical Oncology, Nepean Hospital, Kingswood, NSW Australia; 5 Department of Gynaecological Oncology, Westmead Hospital, Sydney, NSW Australia; 6 Garvan Institute for Medical Research, Darlinghurst, Sydney, NSW Australia; 7 Peter MacCallum Cancer Center, Melbourne, VIC Australia; 8 Sir Peter MacCallum Department of Oncology, The University of Melbourne, Parkvile, VIC Australia; 9 The Daffodil Centre, The University of Sydney, a joint venture with Cancer Council NSW, Sydney, NSW Australia

**Keywords:** Cancer, Molecular medicine, Oncology

## Abstract

Improved biomarkers of treatment response are needed for patients with high-grade serous ovarian cancer (HGSC). A challenge is substantial anatomical site-to-site variation in expression. We completed data-independent acquisition–mass spectrometry (DIA-MS) analysis of 404 fresh frozen and 78 formalin-fixed, paraffin-embedded HGSC tissue samples from the ovary (adnexal) and a common secondary site (omentum) in 11 patients. This was compared with mutation testing, gene expression, and whole-genome copy number profiling. Proteins with relatively stable intra- and variable inter-individual expression (*n* = 1651), included a 52-protein module reflecting interferon-mediated tissue inflammation, indicative of a cGAS-STING pathway cytosolic double-stranded (ds) DNA response. The dsDNA sensing/inflammation score was higher in the omentum compared with the ovary. Ovarian HGSC samples showed marked inter-individual differences in inflammatory and immune responses to DNA damage. Stable discriminative features of the HGSC proteome, a prerequisite for clinical predictive biomarkers, are detectable in ovary (adnexal) tissue samples.

## Introduction

High-grade serous ovarian cancer (HGSC) has a disproportionate impact on cancer-related mortality in women. Currently, the overall five-year survival for HGSC is around 45%, representing only modest improvement over several decades^1^.

Platinum-based chemotherapy has been a mainstay of ovarian cancer treatment for many years. A molecular basis for the effectiveness of this agent emerged from evidence showing that defects in the tumor suppressor genes *BRCA1* and *BRCA2*, which are prevalent in ovarian cancer, cause homologous recombination DNA repair (HR)-deficiency in cancer cells, and increased sensitivity to the cytotoxic effects of platinum compounds. This molecular vulnerability is further exploited by synthetic lethal inhibition of PARP1/2 with a range of PARP inhibitor (PARPi) agents as maintenance in first-line treatment, and at relapse, showing substantial benefit in patients with HR-deficient ovarian cancer due to *BRCA1/2* mutations or other genetic lesions, estimated to be over 50% of HGSC^2,3^.

There is an urgent need to improve treatment options for HGSC. This includes better selection of patients who will benefit from existing treatments, including HR-deficiency targeted therapy, and discovery of new targeted therapeutic options. Reliable tissue-based predictive biomarkers that can guide treatment selection are key to progress in this area. A challenge for their definition is the large volume of disease that is characteristically present at diagnosis in patients with HGSC, which can show considerable heterogeneity within and between anatomical sites^4,5^. This is exemplified by difficulty assigning individual cases to one of the four gene expression-based molecular subtypes of HGSC on the basis of multi-analyte mRNA assays, that impedes clinical application of this classification^6–8^.

The development of high-sensitivity mass spectrometry (MS) based proteomic analysis that is applicable to biopsy-sized tissue samples has potential to progress the search for novel predictive biomarkers^9^. However, since only a small sample of a large cancer is ever analyzed, spatial variation in the cancer tissue proteome, and the potential impact of limited tissue sampling must be factored into discovery phase biomarker research^10^.

We have completed a detailed study of the HGSC proteome in 11 individuals by comparing genomic and gene expression features with 482 proteomic results derived from fresh frozen (FF) or formalin-fixed paraffin-embedded (FFPE) samples from both the ovary and a common metastatic site, omentum. The aim was to identify stably expressed proteins or protein signatures to form a basis for reliable clinical indicators. Our findings gave emphasis to the influence of the sample site of origin on biomarker expression and revealed opportunities to detect mechanisms shaping the HGSC tumor immune microenvironment (TIME) from tissue proteomics. The dataset also forms a useful resource to investigate molecular heterogeneity in HGSC.

## Results

**Fig. 1:**
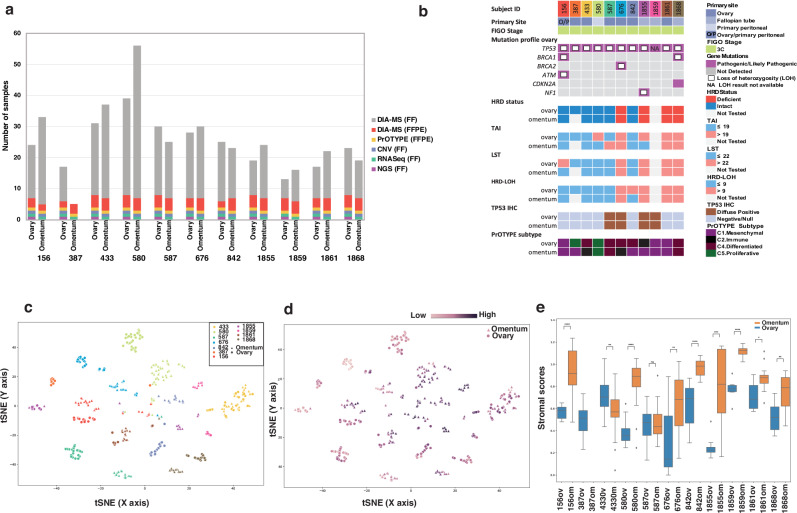
Multi-omic analysis of HGSC in ovary and omentum from 11 individuals. **a** The cohort included 11 individuals with HGSC. Tumor tissue from both the ovary/adnexal site and omentum was extensively sampled and analyzed. The final dataset included DIA-MS proteomic analysis of multiple biopsy-sized samples of FF tissue (*n* = 404, 6–49 per tissue, 11–80 per individual), and FFPE tissue sections (*n* = 78, 2–4 per tissue, 5–8 per individual). Additional data were multigene targeted next generation sequencing (NGS) mutation analysis (FF ovary), whole-genome single nucleotide polymorphism (SNP) microarray copy number variation (CNV) analysis (FF ovary and omentum), whole-genome gene expression (RNASeq) (FF ovary and omentum) and gene expression molecular subtype (NanoString PrOTYPE) analysis (FFPE ovary and omentum). **b** Summary of phenotypic and genotypic features. TAI telomeric allelic imbalance (TAI), LST large-scale transitions, HRD-LOH loss of heterozygosity, IHC immunohistochemistry. **c** The global proteomic landscape of 404 individual FF samples was influenced by inter-individual differences (tSNE) (proteins *n* = 7232). **d** The same plot colored by relative stromal score showed similarity between cases with high stromal content from disparate individuals. **e** Distribution of stromal scores in individual FF samples taken from ovary and omentum. The range of scores from a single piece of tissue was variable reflecting heterogeneity in tissue content. Stromal scores tended to be higher in omentum samples, and the difference was significant in eight out of ten cases (*n* = 404) (*t* test, *p* < 0.05).

Tumor tissue from 11 individuals with HGSC was extensively characterized by multi-parameter profiling: next-generation sequencing (NGS) panel mutation testing, whole-genome gene copy number variation (CNV), whole-transcriptome analysis (RNA-Seq), HGSC molecular subtype (PrOTYPE) and intensive multi-sample proteomic analysis (DIA-MS) of more than 400 individual samples (Fig. 1a). The patients were representative of the most common presentation for epithelial ovarian cancer, all were diagnosed with FIGO Stage IIIC HGSC, with a median age of 62 (range 41 – 74) (Supplementary Table 1). *TP53* mutations were identified in all cases, and there were pathogenic *BRCA1/2* variants in three (3/11, 27%) (Fig. 1b, Supplementary Table 2).

### The global proteomic landscape was shaped by inter-individual differences and tissue content

Proteomic profiles of 404 FF samples from the ovary (adnexa) and one of the most common metastatic sites (omentum) from each patient (11-80 samples per individual), were included in the final analysis (Supplementary Fig. 1). Proteomic profiles included 7232 individual proteins quantified in at least one sample, with a median of 5299 proteins across all samples (Supplementary Fig. 2a, b, Supplementary Table 3).

The global proteomic landscape showed closest similarity between samples taken from the same piece of tissue. Samples from the ovary and omentum in one individual were generally more similar to each other than to samples from the same site in another person (Fig. 1c). However, the relative contribution of non-cancer cell elements was influential. A stromal score derived from 20 proteins that were common between stroma-rich samples (see Methods), and the ESTIMATE stromal signature^11^, demonstrated that high stromal content could dominate the inter-individual differences in the proteome (Fig. 1d). The stromal score was significantly higher in omentum than matched ovarian tumor samples in 8/10 cases (Fig. 1e).

### Stably expressed proteins reflect clinically relevant features of HGSC

**Fig. 2:**
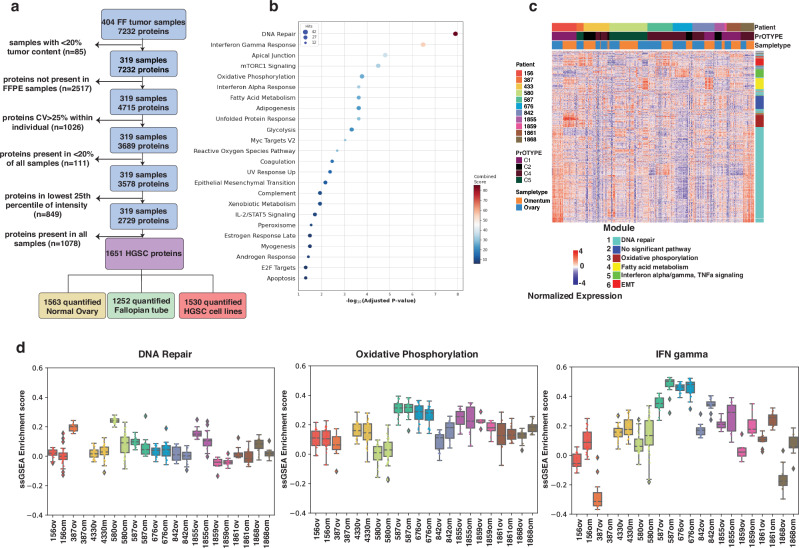
Inflammatory response is characteristic in the HGSC proteome. **a** Filtering steps to identify stable intra-individual and variable inter-individual expression. **b** Biological pathways represented by proteins in the final protein matrix. Dot plot showing enriched hallmark pathways (adj *p* < 0.05, Fisher exact) using gene set enrichment. The size of circle corresponds to number of genes. The combined score is natural log of the p value multiplied by the z-score, where the z-score is the deviation from the expected rank^48^. **c** WGCNA identified six protein co-expression modules from the 1648 mapped stable discriminative proteins in 319 FF tissue samples. Different modules gave representation to DNA repair (module 1, aqua), oxidative phosphorylation (module 3, brown), fatty acid metabolism (module 4, yellow), interferon α (IFNα) and IFNɣ signaling (module 5, green) and Epithelial Mesenchymal Transition (EMT) (module 6, red). Red-blue spectrum represents relative protein abundance (*z* score normalized protein expression). **d** The range of ssGSEA scores derived from expression of distinct pathway related proteins in modules 1 (aqua), 3 (brown) and 5 (green) respectively, across multiple FF samples from a single piece of ovary or omental HGSC tissue (samples *n* = 404, protein matrix *n* = 4715).

Predictive biomarkers with a high degree of variation in different tumor samples from the same patient have limited utility. We therefore focused on identifying proteins with stable expression between multiple samples from one individual, and variable expression between individuals (stable discriminative proteins). To do this, a series of qualification steps was applied to the FF protein matrix, including the requirement for proteins to be detected in both FF and FFPE tissues, show limited variation between multiple samples from the same individual (Coefficient of Variation (CV) < 25%), and non-uniform detection across the cohort to avoid inclusion of housekeeping proteins (Fig. 2a). This resulted in a matrix of 1651 proteins (1648 mapped to known genes), of which 1563 (95%) and 1252 (75.8%) were also detected in samples of normal ovary and normal fallopian tube respectively (Supplementary Table 4), and 1530 (92.5%) were identified in two patient-derived HGSC cell lines. Hallmark pathways represented by these proteins included DNA Repair, oxidative phosphorylation, Interferon (IFN) γ and IFNα response, mammalian target of rapamycin complex 1 (mTORC1) signaling, and oxidative phosphorylation (Fig. 2b).

Weighted correlation network analysis (WGCNA) of the 1648 mapped stable discriminative proteins identified six co-expressed modules that were enriched for distinct pathways (Fig. 2c). Scores derived from single sample gene set enrichment analysis (ssGSEA) for DNA Repair drawn from module 1, and oxidative phosphorylation drawn from module 3 in a 4715 protein matrix, showed a limited dynamic range with inter-sample variation largely obscured by intra-sample differences (Fig. 2d). In contrast, scores reflecting IFNɣ-related pathway proteins from module 5 extended over a larger range, allowing samples to be more readily distinguished.

### Inflammation associated with cGAS-STING pathway activation is a stable feature in the HGSC tissue proteome

**Fig. 3:**
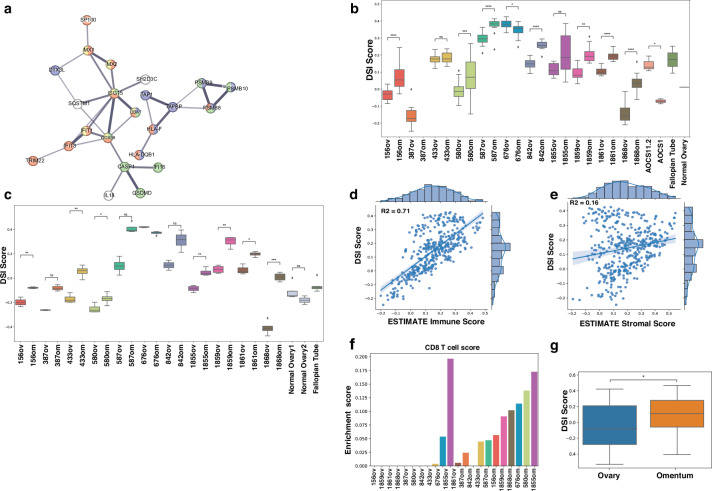
Expression of inflammatory proteins and immune cell infiltrates are different in primary and omental tissues. **a** STRING network of 23/52 core connected proteins DSI proteins^45^. Nodes colored by Reactome pathways: Interferon signaling (red) [Reactome stable identifier R-HSA-913531], Innate immune system (green) [R-HAS-168249], adaptive immune system (purple) [R-HAS-1280218], ISG15 antiviral mechanism (yellow) [R-HSA-1169408]^49^. **b** The range of DSI scores across multiple FF tissue samples from primary and omental tissues (samples *n* = 404, 6–49 samples per tissue), normal fallopian tube tissue (two samples), a pooled normal ovarian tissue sample, two HGSC cell lines (three samples per cell line) (protein matrix *n* = 4715). Scores were significantly higher in omental samples in seven individuals (*t* test independent *: 1.00e-02 < *p* ≤ 5.00e-02, **: 1.00e-03 < *p* ≤ 1.00e-02, ***: 1.00e-04 < *p* ≤ 1.00e-03, ****: *p* ≤ 1.00e-04). **c** The range of DSI scores across multiple FFPE HGSC tissue sections from ovary (*n* = 37) and omental tissues (*n* = 41) (2–4 per tissue), fallopian tube tissue (*n* = 5), normal ovarian tissue (*n* = 6) (protein matrix *n* = 4715). **d** DSI and ESTIMATE immune scores were positively correlated (*n* = 404, *R*^2^ = 0.72, Pearson correlation)^11^. **e** There was no significant correlation between the DSI and ESTIMATE stromal scores (*n* = 404, *R*^2^ = 0.16, Pearson correlation)^11^. **f** CD8+ T cell infiltration scores derived from CIBERSORTx analysis of RNA-Seq data^13^. Only two ovary samples showed appreciable CD8+ T-cell infiltration. **g** The DSI score was significantly higher in HGSC samples taken from omentum (*n* = 38) compared with samples from ovary (*n* = 40) in an independent published proteomic study^14^ (*: *p* < 0.05, *T* test).

Module 5 comprised 52 proteins with an inter-connected core network that reflected tissue inflammation associated with type I and type II interferon mediated innate immune responses (e.g. BST2, CASP1, CMPK2, IFI35, IFIT3, ISG15, MX1, PSM8, PSM9, TAP1), and activation of the cGAS-STING cytosolic double stranded (ds)DNA sensing pathway (e.g. CASP1, DDX58, IL18, IFI16). Antigen processing and presentation through MHCI (HLA-F, TAP1, TAPBP) and MHCII (HLA-DQB1, HLA-DRB3) pathways was also represented (Fig. 3a). Co-expression of these proteins is consistent with gene expression-based DNA sensing/interferon gene expression signatures that have been described in HGSC and increasing evidence that cytosolic dsDNA sensing causes tissue inflammation and immune activation in the HGSC tumor microenvironment^12^. We refer to the ssGSEA score representing module 5 protein expression in each sample as the dsDNA sensing/inflammation (DSI) score.

### Inflammatory signaling and immune cell infiltration are higher in omental tissue samples

DSI scores were consistent between multiple samples from a single piece of tissue in most cases (Fig. 3b, c). However, a striking finding was that scores were generally higher in samples taken from omentum compared with the primary site and this difference was statistically significant for FF samples from 7/10 individuals. Scores for normal fallopian tube, ovarian tissue and two HGCS cell lines were within the range of cancer samples. We found a strong positive correlation between DSI and ESTIMATE immune scores (R^2^ = 0.71, Pearson correlation) (Fig. 3d)^11^. There was no association with stromal scores (R^2^ = 0.16, Pearson correlation) (Fig. 3e)^11^, indicating independence of the DSI score from the stromal content of tissue samples.

The relative abundance of immune cell types in ovarian and omentum samples was examined by CIBERSORTx analysis of RNA-Seq data^13^. Overall, CD8 + T cell scores were higher in omentum samples, with only two cases (676 and 1855) showing appreciable scores in the ovarian sample (Fig. 3f). The macrophage profile was also different between ovarian and omental samples with M0 macrophage scores higher in the ovary, in contrast to M1 and M2 scores that were generally higher in omentum, although these differences was not statistically significant (Supplementary Fig. 3a).

Consistent with these findings, in an independent proteomic study that included prospectively collected samples of HGSC, our DSI score was significantly higher in unmatched samples taken from the omentum (*n* = 38) compared with samples from ovary (*n* = 40)^14^ (Fig. 3g). Although not statistically significant, CIBERSORTx analysis of RNA Seq data also showed higher CD8 + T cell scores in samples from omentum and a skewing of macrophage profiles to the M2 phenotype^14^ (Supplementary Fig. 3b).

### HR-deficiency is associated with tissue inflammation

Assays of HR-deficiency based on characteristic genomic changes that encompass a spectrum of underlying HR defects are evolving as useful clinical indicators^15^. To identify HR-deficiency in this study, a genomic instability score was derived from whole-genome SNP array data in 19 samples from 10 individuals (Supplementary Table 5). Marked intra- and inter-individual variation in DNA copy number profiles was apparent (Supplementary Fig. 4). However, assignment of HR-deficient or HR-intact was concordant between matched tumor samples from individual patients, and eight samples from four individuals were classified as HR-deficient (Fig. 1b).

**Fig. 4:**
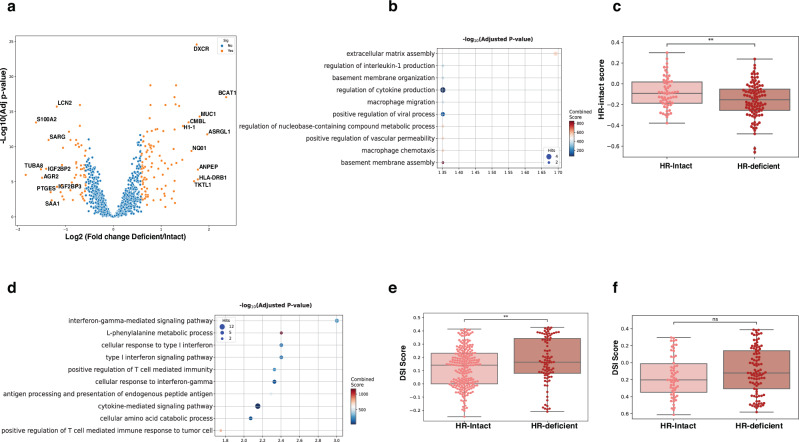
Module 5 proteins are elevated in HR-deficient HGSC. **a** There were 158 differentially expressed proteins between FF samples (≥20% tumor content) from HR-intact (*n* = 220 samples, 6 cases) and HR-deficient (*n* = 90 samples, 4 cases) HGSC (*p* < 0.05, Limma *t* test). **b** Biological processes enriched in proteins that were differentially abundant in HR-intact samples (*p* value < 0.05, Fisher’s exact test). **c** Scores for *n* = 53 HR-intact proteins in HR-intact (*n* = 65) and HR-deficient (*n* = 118) HGSC cases from independent published data^16,17^ (protein matrix *n* = 9600) (*t* test, *p* < =0.02). **d** Biological processes enriched in proteins that were differentially abundant in HR-deficient samples (*p* value < 0.05, Fisher’s exact test). **e** DSI scores in individual FF samples taken from HGSC that were HR-intact (*n* = 264 samples) and HR-deficient (*n* = 124 samples) (independent *t* test, *p* < 0.05). **f** DSI scores for HR-intact (*n* = 65) and HR-deficient (*n* = 118) samples from independent published data^16,17^ (available DSI proteins *n* = 50).

There were 158/1649 mapped stable discriminative proteins differentially expressed between samples from HR-deficient and HR-intact HGSC (Fig. 4a). Proteins (*n* = 57) that were expressed at high level in HR-intact samples included representation of extracellular matrix assembly (Fig. 4b). We examined 53 available HR-intact proteins as a score by ssGSEA in independent published data from 183 cases, and found that scores were significantly higher in HGSC cases classified as HR-intact by genome instability score^16,17^ (Fig. 4c).

Proteins (n = 101) that were differentially abundant in samples from HR-deficient cases gave representation to type I and II interferon mediated inflammation (Fig. 4d), and there were 10 proteins in common with DSI proteins (ISG15, DDX58, CNTRL, DAPL1, HLA-DQB1, HLA-DRB3, HLA-F, MUC1, MX1, PFKP). Consistent with this, the DSI score was significantly higher in HR-deficient samples (Fig. 4e). The DSI score (50 available proteins) was also higher in HR-deficient cases from the independent published series although the difference was not statistically significant^16,17^ (Fig. 4f).

These results are consistent with evidence showing genomic derangement caused by HR-deficiency leads to tissue inflammation via a cGAS-STING pathway mediated innate immune response^12^.

### There is marked inter-individual variation in profiles of DNA damage, sensing and response in primary HGSC tissues

**Fig. 5:**
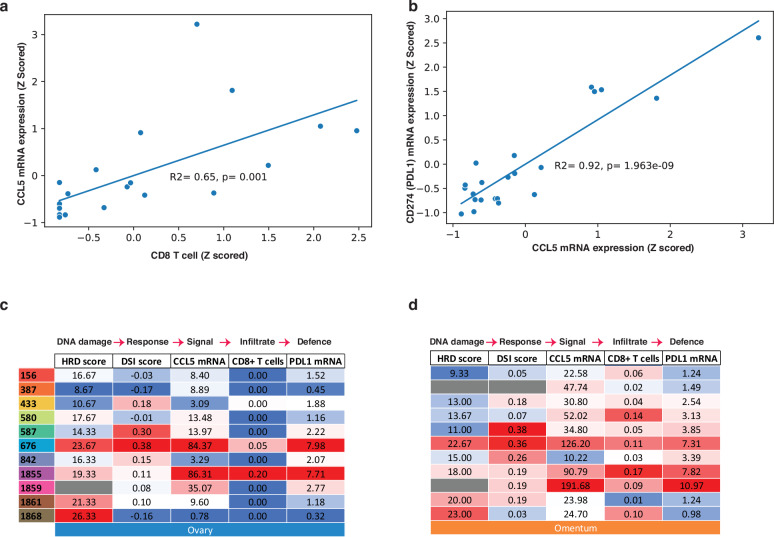
Inflammatory markers are variable in ovarian HGSC tissue, and different from omentum. **a** Correlation between CIBERSORTx CD8+ T cells scores and *CCL5* mRNA expression in RNA-Seq data (*n* = 22, Pearson correlation, *R*^2^ = 0.65)^13^. **b** Correlation between *CCL5* and *CD274 (PDL1)* mRNA expression in RNA-Seq data (*n* = 22, Pearson correlation *R*^2^ = 0.92). **c**, **d** HR-deficiency, DSI scores, *CCL5* mRNA, CD8+ T cell CIBERSORTx scores and *CD274* (*PDL1*) mRNA in ovarian and omental tumor tissues. Color scale indicates position in range.

Chemokine (C-C motif) ligand 5 (CCL5) is a critical intermediate between inflammatory signaling caused by cytosolic dsDNA sensing, and tissue infiltration by CD8+ lymphocytes^12^. Consistent with this, we found a positive correlation between *CCL5* mRNA levels and CD8 + T cell content estimates in HGSC tissues (R^2^ = 0.65) (Fig. 5a). There was also a strong positive correlation between *CCL5* mRNA levels and expression of the immune checkpoint *CD274* (*PDL1*) (R^2^ = 0.92), indicative of an immune evasion response (Fig. 5b).

In samples from the ovary, individual cases of HGSC showed distinct patterns in the sequence of: 1) DNA damage (HR-deficiency), 2) DNA damage response (DSI score), 3) cytokine signaling (*CCL5 mRNA*), 4) CD8+ lymphocyte infiltration and 5) immune checkpoint (*PDL1*) expression (Fig. 5c). All scores were relatively elevated in two HR-deficient cases (676ov and 1855ov). In contrast, HR-deficient cases 1861ov and 1868ov showed low *CCL5* and CD8+ lymphocytes despite an intermediate DSI score in 1861ov. HR-intact case 587ov, with an especially high DSI score, showed low *CCL5*.

In contrast, omentum samples tended to show elevation of all inflammatory markers, with a notable exception of 842om that lacked *CCL5* (Fig. 5d). Overall, these findings suggest that the intrinsic inflammatory environment of omental tissue obscures inter-individual differences in DNA damage response.

## Discussion

The extreme degree of genomic instability in HGSC gives rise to substantial molecular and phenotypic heterogeneity that makes it challenging to identify robust predictive biomarkers and new treatment targets^5^. To address the difficulty of defining reliable tissue-based proteomic biomarkers in HGSC, we took the approach of examining the degree of variation between individual biopsy-sized samples from a single piece of tissue, between ovary and omentum, and between individuals. After filtering the protein matrix to emphasize stable features that were discriminative between individuals, we identified tissue inflammation attributable to cytosolic dsDNA sensing via the cGAS-STING pathway as a relatively robust characteristic.

An intrinsic inflammatory response to cytosolic dsDNA is increasingly recognized as an important influence shaping the TIME of cancers with a high degree of chromosomal instability (CIN), including HGSC^18^. It is associated with the formation of micronuclei consequent to chromosomal mis-segregation in replicating cancer cells^19^; a phenomenon that is exacerbated by DNA-damaging treatments including PARPi^12^. Free cytosolic dsDNA released from ruptured micronuclei can lead to activation of the cGAS-STING pathway, causing stimulation of IFN-I and NFkB-mediated inflammatory transcriptional programs^12,19^. At the tissue level, this can have a profound influence on the balance of immune activation and immune suppressive features, including the profile of immune cells present and expression of immune checkpoint proteins^12,18^.

Our finding of high DSI scores associated with HR-deficiency in HGSC is consistent with this evidence since genomic disruption is exacerbated in HR-deficient cases. It is also consistent with a study showing an association between HR-deficiency and an overlapping gene expression signature in HGSC epithelial cells, which was also associated with expression of *MHCI* and *MHCII* genes^20^. Our further observation that DSI scores were relatively stable across multiple samples supports DSI as a tissue permeative characteristic that is amenable to reproducible measurement.

There has been intense interest in the use of immune checkpoint inhibitor (ICI) therapy for HGSC following the transformative impact in some poor-prognosis cancer types, and evidence of benefit in triple negative breast cancer that shares important molecular characteristics with HGSC^21^. Correlative evidence suggests that cGAS-STING pathway activation could be a relevant biomarker. For example, cGAS-STING activation is associated with CD8+ T-cell infiltration in HGSC tissues and PDL1 expression^12^, which are both predictive indicators of ICI response^22^. However, despite features generally indicative of immunogenicity in HGSC, response rates to ICI treatments are poor^23^.

An explanation is that cancer cells with high levels of CIN, and consequent genomic disruption, are adapted to grow in a chronically inflamed tissue environment^19^. In addition to expression of immune checkpoint proteins, this adaptation can involve clonal or sub-clonal deletion of HLA protein-encoding genes^5^, and deletion or hypermethylation of inflammatory intermediates, including *CCL*5^12^. Moreover, evidence for preferential activation of the immune-suppressive non-canonical NFkB pathway downstream of cGAS-STING has been shown in some cancers with CIN, and is associated with reduced survival^24^.

The relative contribution of different immune evasion mechanisms is attributable to somatic genomic changes that are unique to each HGSC case, and different between disease sites in an individual, contributing to complex and highly variable phenotypes that are difficult to classify or effectively target. This extreme variation was apparent in DNA copy number profiles from our cohort of 11 cases and reflected in phenotypic variability, with only two HR-deficient cases showing appreciable CD8+ T-cell infiltration in tumor tissue from the ovary/adnexal site despite relative elevation of DSI scores in more than half of the cases.

A finding with important practical implications in our study was major differences in TIME profiles between samples taken from the ovary and omentum, with omental tissues showing high inflammatory indicators and stromal scores and skewing of macrophage profiles to the immunosuppressive M2 type. Our findings align with a recent report demonstrating a relative reduction in T cells at the adnexal (primary) site and fewer interactions between PDL1-positive cancer cells and immune cells in the omentum^20^. The omentum is a preferential site of HGSC metastasis and a common site of HGSC tissue sampling for diagnostic purposes or research. Our results show that samples from the ovary and omentum are not comparable for evaluation of mesenchymal or immune features and emphasize the importance of sample site for interpretation of biomarkers.

The main limitation of our study is that the cohort included only 11 cases, which may reduce the generalizability of our findings and preclude meaningful comparison with clinical endpoints. Sampling cancer tissue deposits from a broader range of anatomical sites, and at disease progression or relapse, was beyond the scope of our study, but is needed to examine the stability of potential HGSC tissue biomarkers in more detail. Our approach of coring FF tissues to obtain samples for proteomic analysis limited direct comparison of proteomic and histopathologic features. A combination of proteomics with digital histopathology image analysis, or single-cell tissue analysis techniques, would bring additional insight.

Arguably, the most successful targeted therapy for HGSC to date is PARPi treatment that uses a synthetic lethal strategy to accentuate DNA repair limitations in HR-deficient cancer. In view of the considerable intra-individual heterogeneity that is commonly present in HGSC, a continued strategy of targeting mechanisms that broadly influence disease phenotype may have the greatest utility. Further human tissue-based research is needed to elucidate the potential clinical implications of cGAS-STING activation, for both HR-deficient and HR-intact HGSC in the context of cytotoxic chemotherapy and targeted treatments including PARPi. Our finding of a relatively stable proteomic signature reflecting cGAS-STING activation in HGSC tissues presents options to support these studies.

## Methods

### Patient materials

The cohort included 11 women with HGSC diagnosed between 1992 and 2016. In this study, all cases were designated HGSC, accepting that disease may have originated from fallopian tube, ovary, or peritoneum by current criteria^25^. HGSC tissues were collected pre-chemotherapy treatment. Clinical features are summarized in Supplementary Table 1. Two patient-derived HGSC cell lines were supplied from the Australian Ovarian Cancer Study (AOCS): AOCS 11.2 and AOCS1.

All tissue samples were accessed from the Gynecological Oncology Biobank (GynBiobank) at Westmead Hospital, Western Sydney Local Health District (WSLHD). Written informed consent is obtained from tissue donors, and GynBiobank is approved by the WSLHD Human Research Ethics Committee (HREC) (2019/ETH02603). This research was approved by the WSLHD HREC (2019/ETH02043). AOCS was approved by HRECs at the Peter MacCallum Cancer Centre (Project No. 01/60 and 16/161), University of Melbourne, the Queensland Institute of Medical Research, Westmead Hospital, and all other participating hospitals and cancer registries, and participants provided written informed consent. Research was conducted in accordance with the Declaration of Helsinki.

### Sample preparation for molecular analyses

For proteomic analysis, FF HGSC tissue from ovary (*n* = 11) and matched omentum (*n* = 10) was sampled by subdividing two to five full-thickness, 1.5 mm diameter tissue cores taken with a CryoExtract CXT350 (BioStrategy, Broadmeadows, VIC, Australia), into multiple segments of wet-weight 0.4–6.3 mg (Supplementary Fig. 1a). Digital whole slide images of surface hematoxylin and eosin (H&E) stained tissue sections taken prior, and subsequent to coring were reviewed to record features including an estimate of the percentage of cancer at the surface of each core (Supplementary Fig. 1b, Supplementary Table 6). FF samples of fallopian tube (*n* = 3) and a pool of multiple samples of normal ovary tissue from two respective non-cancer patients, were processed with FF HGSC tissue samples (Supplementary Fig. 1c).

FF tissues were processed with lysis and digestion assisted by pressure cycling technology as previously described^26^, with the exception that samples were cleaned up with C18 solid phase extraction (SPE) after digestion. Peptides extracted from normal ovary tissue samples were pooled to form a control sample.

FFPE tissue blocks of HGSC in the ovary (*n* = 11) and omentum (*n* = 11) were separately processed for proteomics with four 10 µm sections sampled from each block for HGSC and four 20 µm sections for normal tissue. H&E-stained sections were reviewed to select blocks with crude tumor estimates of 20–90% (median 80%) for ovary, and 10–90% (median 40%) for omentum. Samples of FFPE ovary and fallopian tube tissue from the same two non-cancer patients with FF sample analyzed, were processed with FFPE samples. FFPE tissues were pre-processed using the method described by Jullig et al.^27^, and then processed as for FF tissues with the exception that the lysis step was performed at 70 °C.

Cell lines were maintained in RPMI 1640 medium supplemented with 10% fetal bovine serum and 2 mM glutamine. The cell lines were routinely tested, and shown to be free of *Mycoplasma*, and were authenticated against germline DNA by short tandem repeat (STR) profiling (GenePrint®, Promega, Madison, WI, USA). For proteomic analysis, cells were harvested from 150 cm^2^ flasks at ~80% confluency (minimum of 1 × 10^6^ cells) by washing in ice-cold Dulbecco’s phosphate-buffered saline (PBS), gentle cell scraping on ice in cold PBS, and centrifugation (5000 × *g*, 5 min, 4 °C). Three cell pellets were then snap-frozen in liquid nitrogen and stored at −80 °C. Cell line samples were prepared using the Accelerated Barocycler Lysis and Extraction (ABLE) method as previously described^26^ .

FF HGSC tissue was sampled from ovary (*n* = 11) and matched omentum (*n* = 11) for RNA sequencing (RNA-Seq); ovary (*n* = 11) for mutation analysis; and ovary (*n* = 10) and matched omentum (*n* = 9) for whole-genome CNV analysis. Tissue was cryosectioned, and a surface H&E-stained section was reviewed to evaluate tissue content. Either full-face 100 μm cryosections or macrodissected cryosections were processed. Following macrodissection, tumor content of at least 70% was obtained, with the exception of three samples with lower tumor content (20–40%) for RNA-Seq analysis and one sample (30–40%) in the mutation analysis sample set.

FF samples were homogenized using the TissueLyser LT (Qiagen), 30 Hz for 1 min. DNA and RNA were simultaneously isolated using the AllPrep DNA/RNA/miRNA Universal kit (Qiagen) as per manufacturer’s protocol. For DNA purification, pretreatment with RNase A (13 μl of 100 mg/μl) in Buffer P1 (147 μl of 50 mM Tris-HCl pH 8.0, 10 mM EDTA) was performed prior to proteinase K (PK) digestion. DNA was quantified on a Qubit Fluorometer (Thermo Fisher Scientific, MA, USA) and RNA on a NanoDrop spectrophotometer (Thermo Fisher Scientific, MA, USA). RNA quality was assessed using the Agilent 2100 DNA Fragment Bioanalyzer system (CA, USA).

For RNA (NanoString) analysis, FFPE specimens of HGSC in ovary (*n* = 11) and omentum (*n* = 11) were sectioned at 10 µm, de-paraffinized using deparaffinization solution and total RNA was processed according to instructions of the Qiagen miRNeasy FFPE kit (Qiagen) with an elongated 56 °C PK digestion time of 45 min.

### Mass spectrometry

Data-independent acquisition mass spectrometry (DIA-MS) was performed as previously described^28^.

FF HGSC samples were included in a single DIA-MS experiment in randomized batches of 15. Normal fallopian tube and cell line samples were included in a single batch. Technical duplicate DIA-MS runs were performed for >97% of samples, and there was a total of 987 DIA-MS sample runs (Supplementary Fig. 1c). FFPE samples of HGSC and normal tissue were run in a separate experiment, including 189 DIA-MS runs (Supplementary Fig. 5a, b). The pooled FF normal ovarian tissue sample and a HEK293T cell line control^26^ were included in each MS run, and 3 FF peptide extracts from the FF experiment were re-run with FFPE samples.

### Spectral library and DIA-MS data processing

An in silico spectral library was created using DIA-NN (version 1.8)^29^ as previously described^30^. The final spectral library constructed from all FF samples contained 10,470 proteins and 82,325 peptides. The final spectral library constructed from FFPE samples contained 6915 proteins and 36,664 peptides. These spectral libraries were used to perform SWATH searches using DIA-NN as previously described^29^. DIA-NN output data were filtered to retain only precursors from proteotypic peptides with Global.*Q*.Value ≤ 0.01.

Quality checks were performed on peptide-level data to filter out low-intensity or outlier samples and peptides from further analysis. For the FF samples, samples where peptide counts were less than 5000 peptides (*n* = 9) were removed in addition to technical replicates with less than 0.8 Pearson correlation (*n* = 102) to ensure highly concordant data between runs (Supplementary Fig. 1c). This resulted in a total of 876 tissue sample DIA-MS runs in the FF experiment. The data were further logged, median normalized, and summarized to protein level using the MaxLFQ^31^. Peptides, which log2(normalized)<0 were removed. Levels in the final protein matrix were the average of technical replicates, where applicable. This resulted in a total of 7232 proteins quantified in at least one sample (Supplementary Table 3).

Similarly, FFPE samples with less than 5000 peptides (*n* = 8) were removed, and technical replicates with poor correlation were filtered (*n* = 19). The final data matrix included 162 FFPE tissue sample DIA-MS results (Supplementary Fig. 5a). Data were median normalized and protein abundances estimated as above. The resulting protein matrix contained a total of 4,912 proteins quantified in at least one sample (Supplementary Table 7).

More than 90% of the proteins quantified in FFPE samples were also quantified in the FF samples.

### Mutation analysis by multigene panel NGS

Unique molecular identifier (UMI) incorporated amplicon-based, multigene targeted sequencing was performed on DNA (total DNA input ~50 ng) isolated from FF tumor tissue to screen for mutations in 30 ovarian cancer-related genes (Supplementary Table 2).

All coding exons and flanking intron junctions of 19 genes were sequenced: *TP53, ARID1A, BRCA1, BRCA2, NF1, PTEN, ATM, RB1, BRIP1, BARD1, MSH6, MSH2, MLH1, CDK12, FBXW7, CHEK2, PALB2, RAD51C,* and *RAD51D*. Two genes were partially covered: *PIK3R1* (all exons covered except exon 1), and *PMS2* (all exons covered with the exception of exon 15, partial coverage of exons 14 & 12). Hot spot regions were sequenced in nine genes–*NRAS* (exon 2 & 3), *KRAS* (exon 2 & 3), *BRAF* (exon 11 & 15), *AKT1* (exon 4), *PIK3CA* (exon 8, 10 & 21), *CTNNB1* (exon 3), *CDKN2A* (exon 2 & 3), *PPP2R1A* (exon 4–9) and *EIF1AX* (exon 1 & 2).

The targeted region was enriched using QIAseq Targeted DNA custom panel (CDHS-33269Z-1195) and DNA libraries were prepared using QIAseq index as per Qiagen QiaSeq Targeted DNA panel handbook 02/20. Uniquely indexed samples were pooled and sequenced on an Illumina MiSeq V3 to generate 2 × 150 bp reads at a sequencing coverage of x7000 reads per base at the Australian Genome Research Facility (AGRF, Sydney NSW).

The sequence analysis pipeline consisted of sequence alignment to human reference genome build; hg19/GRch37. Data were analyzed in accordance with smCounter2, a UMI-based variant caller (https://github.com/qiaseq/smcounter-v2-paper)^32^. Variants were curated in accordance with bioinformatics scores for SIFT and PolyPhen; followed by examination of ClinVar, dbSNP, and IARC *TP53* categorization. Genetic variants were interpreted according to ACMG/AMP guidelines^33,34^ and clinical phenotypes. This test considered somatic point mutations, short insertions, and deletions. Genetic variants of SNV < 4% and INDEL < 20% variant allele frequency were not reported.

### Whole-genome CNV and HR-deficiency score

DNA single-nucleotide polymorphism (SNP) microarray CNV analysis on matched FF tumor from ovary and omentum was performed on Illumina OmniExpress-24 BeadChip arrays, according to manufacturer’s instructions at AGRF. B-allele frequencies (BAF) and log R ratio (LRR) were extracted using Illumina’s GenomeStudio 2.0.4 with Genotyping module 2.0.4 software, using default Illumina settings. Genome alteration print (GAP) analysis was applied to characterize copy number profiles^35^ (Supplementary Fig. 4). LOH profiles of mutated genes were obtained from SNP arrays using the GAP analysis^35^. Gene-level copy number values were derived from linear copy number values obtained from “all_data_by_genes.txt” table from Genomic Identification of Significant Targets in Cancer 2.0 (GISTIC2.0) analysis^36^ (Supplementary Table 8).

A logistic regression model was utilized to predict the HR-deficiency status using genomic lesion scores from whole-genome SNP arrays^37^. The training dataset for the model included genomic lesion scores of primary tumor samples from the AOCS (*n* = 80) with known HR-deficiency status^4^. Three genomic lesion scores, telomeric allelic imbalance (TAI)^38^, loss of heterozygosity (HRD-LOH)^39^, and large-scale transitions (LST)^40^ were calculated by applying the approach described by Marquard et al*.*^16^. The individual genomic lesion scores and the arithmetic mean of the three scores (HRD-mean score) were used as independent variables in the logistic regression model, with HR-deficiency status based on mutation or methylation state of *BRCA1/2* as the binary response variable. The univariate model involving HRD-mean score was determined to be the most significant binary classifier (AUC = 0.8605, *P* < 0.0001) in the prediction of HR-deficiency status and also estimated to have the lowest cross-validation error based on a fourfold cross-validation analysis. The HR-deficiency status in this study was predicted using the univariate classifier based on HRD-mean score (Supplementary Table 5).

### Whole-transcriptome gene expression analysis by RNAseq

RNA from FF ovarian and omentum HGSC tissues (RIN > 9) were used for library preparation using TruSeq Stranded mRNA (Illumina, San Diego, CA) as per manufacturer’s instructions. Libraries were sequenced using NovaSeq 6000 (Illumina) resulting in 150 bp paired-end reads at AGRF.

Initial QC on the raw FASTQ files was done using FastQC (v0.11.8). Reads were trimmed for low-quality bases, adapters, N content, and poly-A tails using fastq-mcf (v1.05). Contamination was checked using FastQ Screen (v0.11.4). Reads were mapped to the human reference GRCh37.92 using the STAR two-pass method (v2.6.0b). Mapped reads were sorted using Picard Tools (v2.17.3). Additional QC after mapping was performed using Picard Tools CollectRnaSeqMetrics (v2.17.3) and RSeQC (v2.6.4). Counts were generated on the ensemble release GRCh37.92 GTF annotation using HTSeq (v0.10.0). Counts were done on the exons only using the “intersection-nonempty” mode (Supplementary Table 9).

### NanoString nCounter gene expression HGSC molecular subtype (PrOTYPE) analysis

Gene expression-based HGSC molecular subtypes were derived from analysis of FPPE samples of ovary (*n* = 11) and omentum (*n* = 11) using the locked–down clinical PrOTYPE **(Pr**edictor of high-grade-serous **O**varian carcinoma molecular sub**TYPE**) assay on the NanoString platform (NanoString, Seattle, WA, USA)^41^ (Supplementary Table 10). The NanoString codesets consist of PrOTYPE genes (55 prediction model gene probes plus five housekeeping genes probes).

RNA Hybridization was performed using the nCounter gene expression XT chemistry protocol. For a 12 nCounter assay, a master mix of the Reporter CodeSet, Capture CodeSet and Hybridization Buffer was prepared with 500 ng of FFPE RNA or 100 ng of FF RNA. Samples were incubated for 20 h at 65 °C in a preheated thermo cycler. Hybridized samples were processed on the nCounter Prep Station and loaded cartridges were scanned at maximum resolution (555 Field of View) on the nCounter Digital Analyser.

Resulting raw data were normalized and HGSC molecular subtypes predicted using the PrOTYPE web-based tool (https://ovcare.shinyapps.io/PrOTYPE/) that returns a prediction probability and a predictive entropy value for each subtype. The entropy value estimates certainty of prediction where 0 entropy corresponds to a near perfect prediction or “pure” subtype, while 2 entropy corresponds to near equal chance of assignment to any subtype.

### TP53 immunohistochemistry (IHC)

IHC analysis was performed on a 4 µm tissue microarray (TMA) section representing all 11 patients in the study cohort. To construct the TMA, duplicate 1 mm cores were taken from matched ovarian and omental FFPE tumors and assembled into a single recipient block using the Galileo TMA CK4500 (ISENET). Staining with heat-induced epitope retrieval (100˚C for 56 min in Ventana Ultra CC1 Solution (Roche Diagnostics, Cat # 950-224)) and p53 (SP5) antibody (Cell Marque™, Cat# CMC45331030, dilution 1:50) was performed using the Ventana Benchmark ULTRA platform. Staining intensity and pattern was evaluated by a three-observer consensus review.

### Data analysis

To visualize the proteomics data, we used t-distributed stochastic neighbor embedding (tSNE), a commonly used dimensionality reduction technique in data visualization. The tSNE algorithm was implemented using the sklearn-tsne (https://scikit-learn.org/stable/modules/generated/sklearn.manifold.TSNE.html) module in python programming language. The tSNE plot was generated by projecting the high-dimensional data onto a two or three-dimensional space, allowing visual inspection of the distribution of data points in a reduced dimensionality. The samples were further colored by various features for visualization.

ssGSEA^42^, which calculates a separate enrichment score for each pairing of sample and a gene set, was applied to proteomic data. The enrichment scores are normalized (NES) for differences in gene set size and in correlations between gene sets and the expression dataset, thus enabling comparison between samples. Each ssGSEA enrichment score represents the degree to which the proteins, in this case, in a particular protein set, are coordinately up- or down-regulated within a sample. The ssGSEA analysis was performed using the GSVA package in R^43^. The DSI score was derived from ssGSEA.

A stromal score was derived from ssGSEA analysis of 20 proteins that were common between proteins that were differentially abundant in samples that comprised 100% stroma by matched histopathology review, and the ESTIMATE stromal signature^11^. Differential expression analysis was performed using the limma R package (http://bioconductor.org/packages/release/bioc/html/limma.html). Stromal signature proteins were ASPN, MXRA5, COL14A1, COL1A2, COL6A3, VSIG4, COL15A1, COMP, F13A1, CILP, PTGIS, ITIH3, LUM, DCN, COL3A1, THBS2, FBLN2, BGN, C1QB and OLFML1.

Multiple filtering steps were applied to identify stable discriminative proteins in FF samples, as depicted in Fig. 2a. The different filtering steps were as follows: Samples with less than 20% tumor were excluded from the analysis. Proteins that were present in the FF but not in the FFPE data matrix were removed. The CV was calculated for each protein across all the samples from a given patient as CV = $\frac{\sigma }{\mu }$, where µ is the mean of protein expression across all samples and σ is the standard deviation. If the CV was less than 25% across all samples from an individual patient, the protein was retained. Proteins quantified in <20% of samples were removed. Proteins in the lowest 25th percentile for intensity were removed. Proteins expressed in all samples were removed. The final list of stable discriminative proteins included 1651 proteins, with 1648 mapped to known genes.

A weighted gene set network analysis (WGCNA) was performed using the subset of mapped stable proteins (n = 1648) using the WGCNA R package^44^. Soft thresholding power was determined using the pickSoftThreshold function. Network was further constructed using optimal power =7, minModuleSize=30, and TomType= ”unsigned”. The resulting modules were colored and annotated using enrichGO and gsePathway modules from clusterProfiler R package.

Protein-protein interaction network illustration and related functional enrichment analyses were performed in STRING (database version 12.0)^45^. Selected parameters were: Network type: physical subnetwork (disconnected nodes removed); Active interaction sources_all; Minimum required interaction score_medium confidence (0.400); Network edges_ confidence (line thickness indicates strength of supporting data).

RNA-Seq was summarized at the gene level and used as an input for CIBERSORT analysis using the CIBERSORTx tool^13^. CIBERSORTx is an analytical tool that estimates abundances of member cell types in a mixed cell population, using gene expression data. A prebuilt 22 immune-cell type signature from CIBERSORTx was used to score the RNA-Seq data and identify the contribution of each cell type to the sample. The default parameters were used. The cell fractions for 22 cell types (B cells naive, B cells memory, Plasma cells, T cells CD8, T cells CD4 naive, T cells CD4 memory resting, T cells CD4 memory activated, T cells follicular helper, T cells regulatory (Tregs), T cells gamma delta, NK cells resting, NK cells activated, Monocytes, Macrophages M0, Macrophages M1, Macrophages M2, Dendritic cells resting, Dendritic cells activated, Mast cells resting, Mast cells activated, Eosinophils, Neutrophils) along with correlation p-values are provided from the analysis. The samples were mapped with clinical metadata and further plotted as box plots using R custom scripts.

Pathway enrichment analysis was performed using the gseapy python package (https://github.com/zqfang/GSEApy). “Kegg_2019_human” and “GO_Biological_Process_2021” as the gene sets. The enriched processes were plotted as dot plot using python.

Heatmaps were generated using Seaborn python package (https://seaborn.pydata.org/) or using GENE-E tool (https://software.broadinstitute.org/GENE-E/). Euclidean distance with average linkage was used for all clustering. The data was z-scored for visualization.

Statistical analyses as noted in the text were performed using python scipy stats package (https://docs.scipy.org/doc/scipy/reference/stats.html). A *t*-test was performed between HR-deficient and HR-intact samples to identify significantly dysregulated proteins (p value < 0.05 and fold-change >2) within the stably expressed proteins. BH corrected adj. *P*-values < 0.05 were considered statistically significant throughout. All statistical tests were two-sided unless otherwise stated.

Volcano plot of dysregulated genes was plotted using the Enhanced Volcano R package (https://bioconductor.org/packages/release/bioc/html/EnhancedVolcano.html).

A Spearman rank correlation analysis was performed to assess the correlation between normalized mRNA expression and immune enrichment scores. Similarly, mRNA data for *CCL5* and PDL1 were normalized to compare with normalized immune scores, module 5 scores, and HR-status.

All boxplots indicate median (center line), upper and lower quartiles (box limits), 1.5× interquartile range (whiskers), and outliers (points).

## Supplementary information

Supplementary information

Supplementary information

Supplementary AOCS Group Members

Supplementary AOCS Group Members

## Data Availability

The mass spectrometry proteomic data have been deposited in the ProteomeXchange Consortium via the PRIDE^46^ partner repository with the dataset identifier PXD042150. RNA-Seq data have been deposited in the National Center for Biotechnology Information (US) Gene Expression Omnibus (GEO)^47^ with the accession GSE277107.
