# Supplementary material for: Overcoming intra-tumoral heterogeneity for biomarker discovery in the high-grade serous ovarian cancer proteome
Source: NPJ Precis Oncol. 2025 Jun 11;9:172. doi: 10.1038/s41698-025-00911-y (PMC12159146; doi:10.1038/s41698-025-00911-y)
Supplement: Supplementary file 1 — Supplementary information [file 41698_2025_911_MOESM1_ESM.pdf]

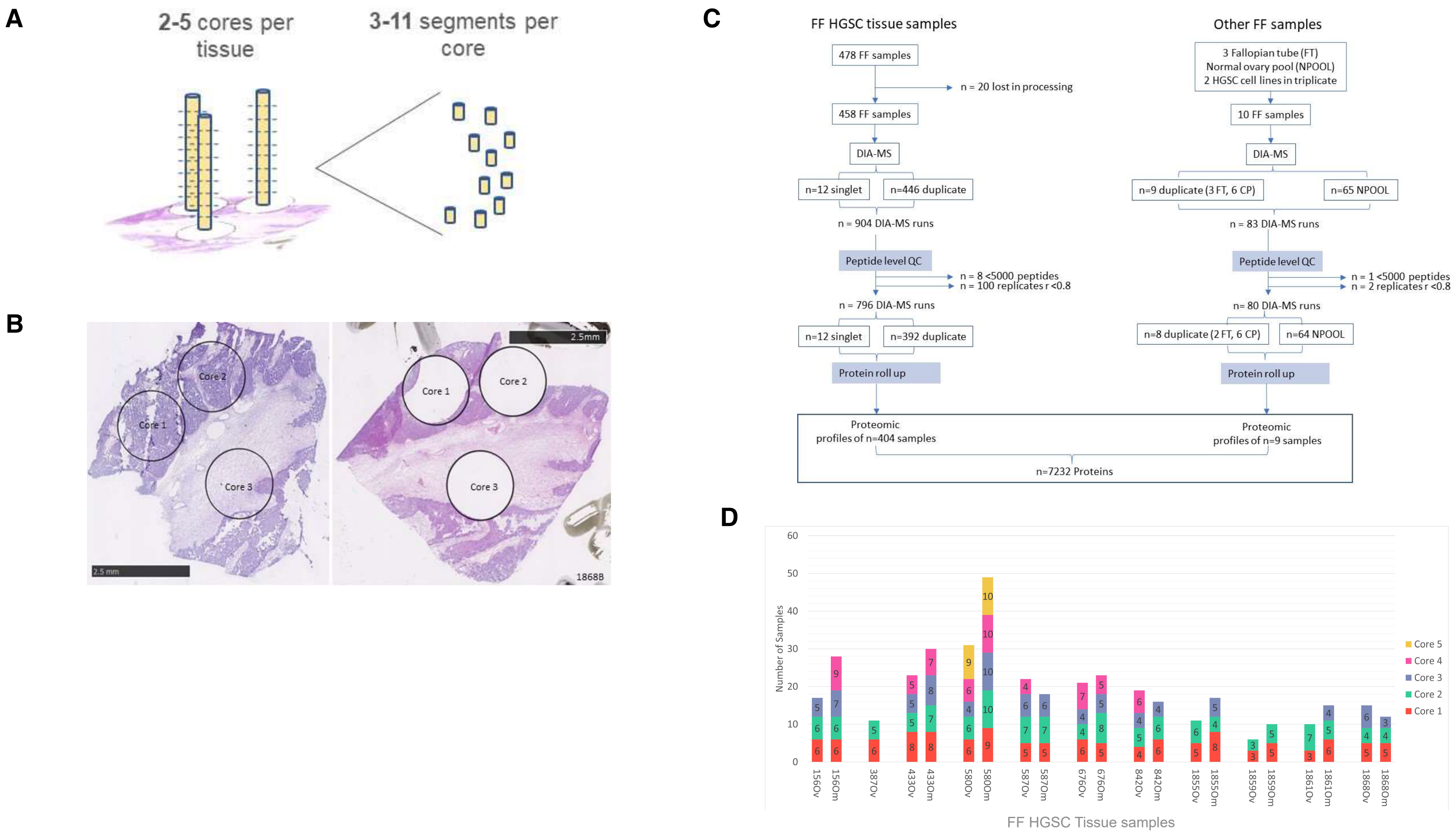

Supplementary Figure 1 - Sampling of fresh frozen (FF) tissue for DIA-MS

A. Multiple full-thickness, 1.5 mm diameter cores were taken from frozen tissue samples. Cores were then divided into segments to obtain individual samples for analysis. There were 2-5 cores per tissue sample, and 3-11 segments per core. This resulted in 7-49 samples per piece of tissue and 14-88 samples per individual.
B. A surface H&E-stained section before and after tissue cores were taken. This allowed an estimate of the % cancer to be recorded.
C. Process flow of individual FF tissue and HGSC cell line samples from sample collection to the final protein matrix. HEK293 cell line control samples were also analysed.
D. The final number of individual FF HGSC samples contributing data to the analysis (n=404).

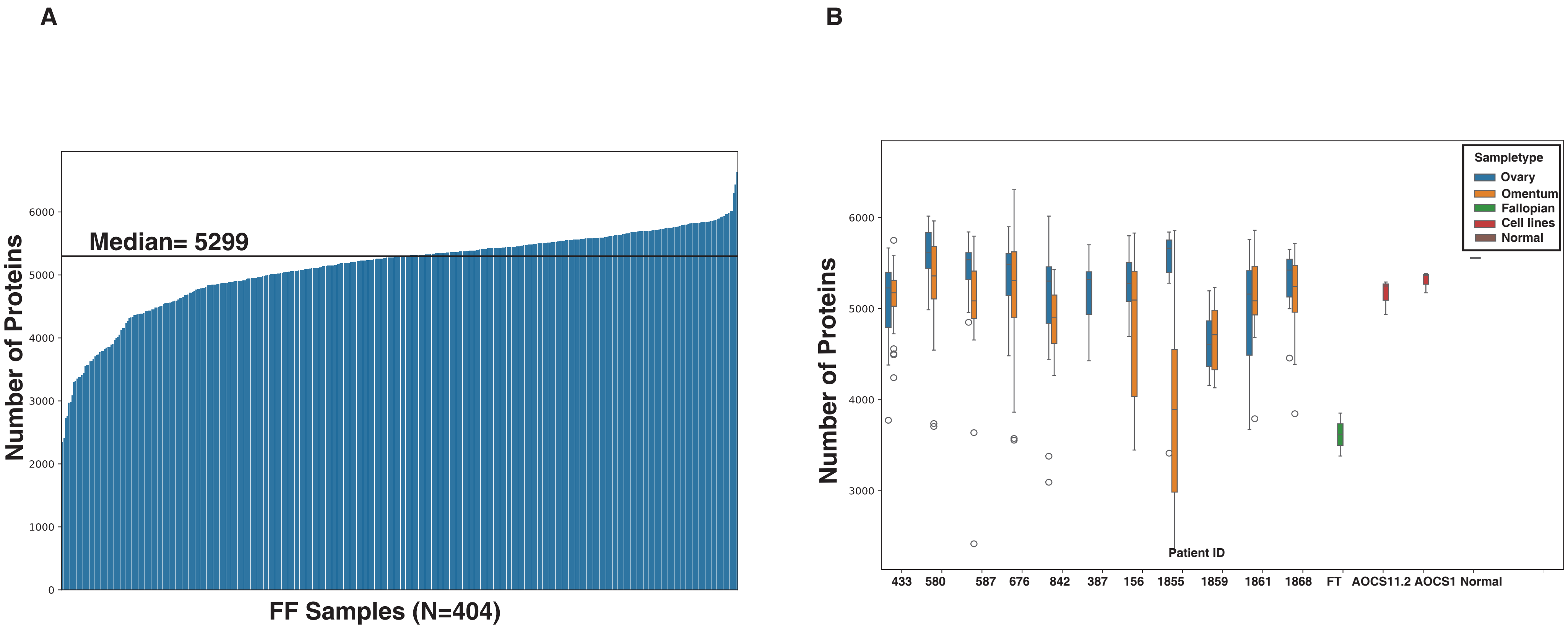

Supplementary Figure 2 – Proteins quantified in FF samples

A. There were 7,232 proteins quantified across 413 FF samples (n=404 HGSC tissue, 2 fallopian tube, 6 cell line and 1 normal ovary pool control). The median number of proteins quantified per sample across the cohort was 5,299.

B. Proteins quantified in each tissue. Normal is normal ovarian tissue pooled sample.

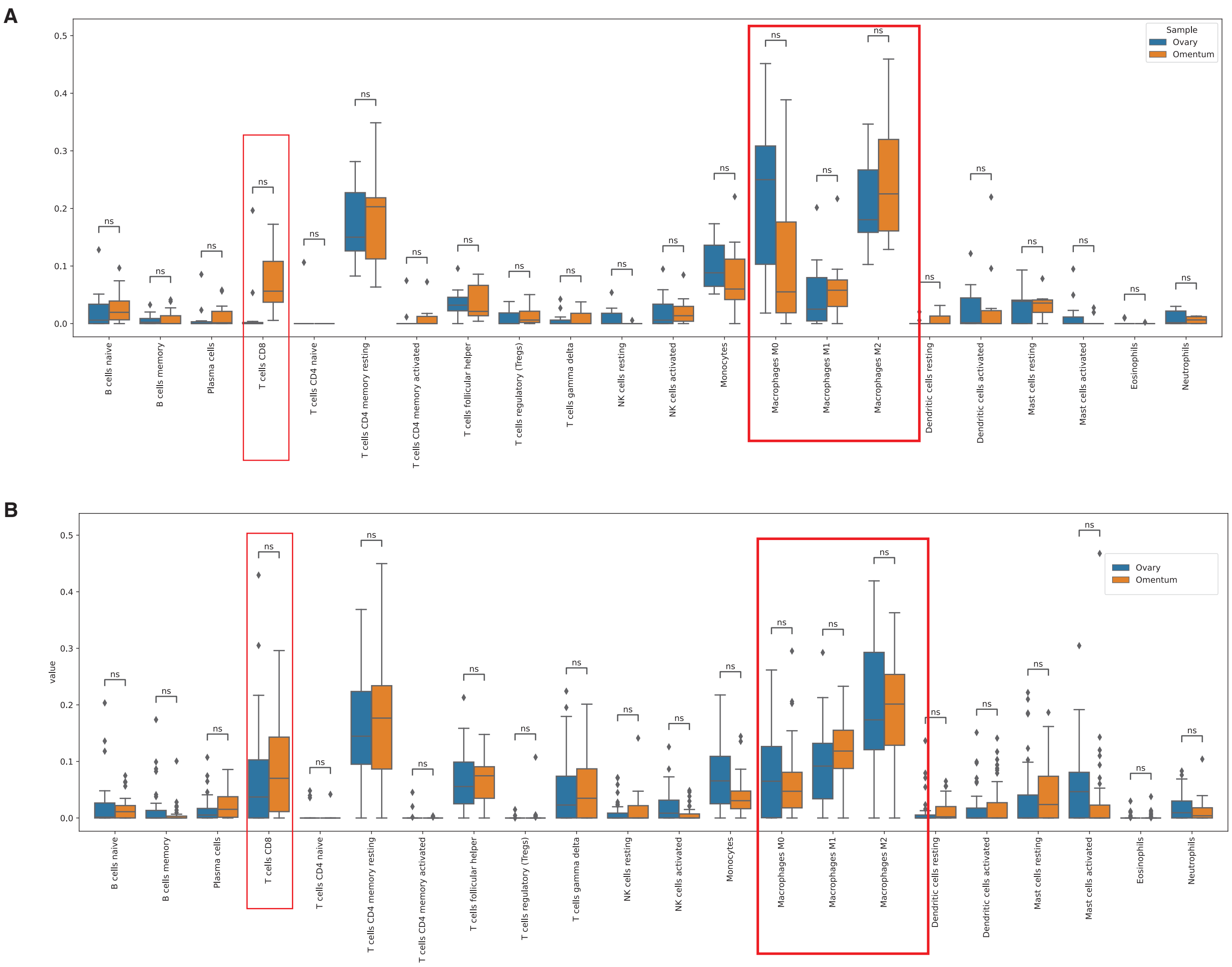

Supplementary Figure 3 – Immune cell infiltrates in ovary and omental tissues

A. Immune cell infiltrate scores profiles across ovary and omental HGSC tissues from CIBERSORTx analysis [1] of RNASEq data in this cohort (n=11) (Mann-Whitney U-test, p<0.05).

B. CIBERSORTx [1] immune cell infiltrate scores from an independent published study that included unmatched HGSC samples from ovary (n=40) and omentum (n=38) [2] (Mann-Whitney U-test, p<0.05).

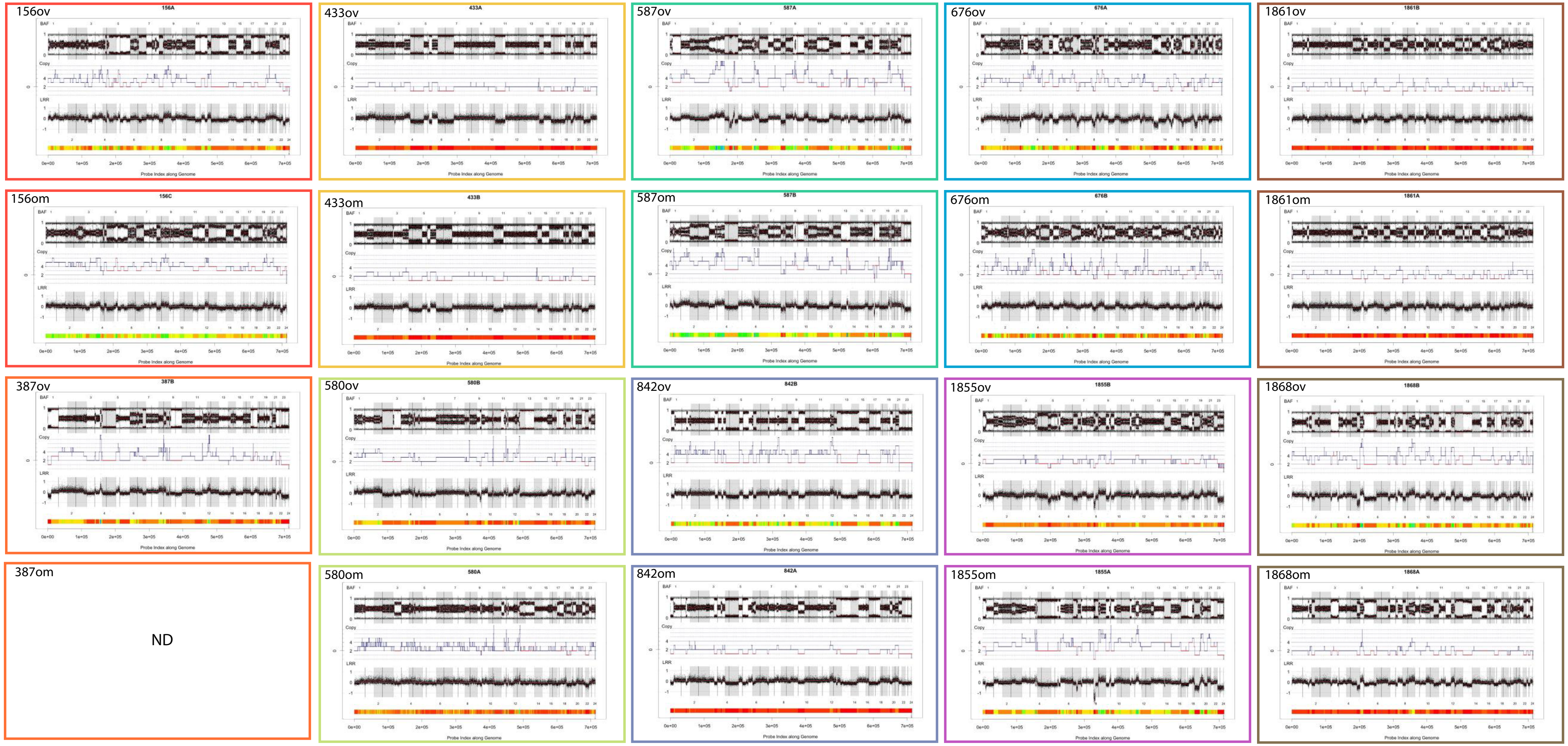

Supplementary Figure 4 – Genomic profiles from SNP array analysis

Genome alteration print (GAP) profiles [3] derived from SNP array analysis of FF tissues. BAF B-allele frequencies, LRR Log R ratios.

A

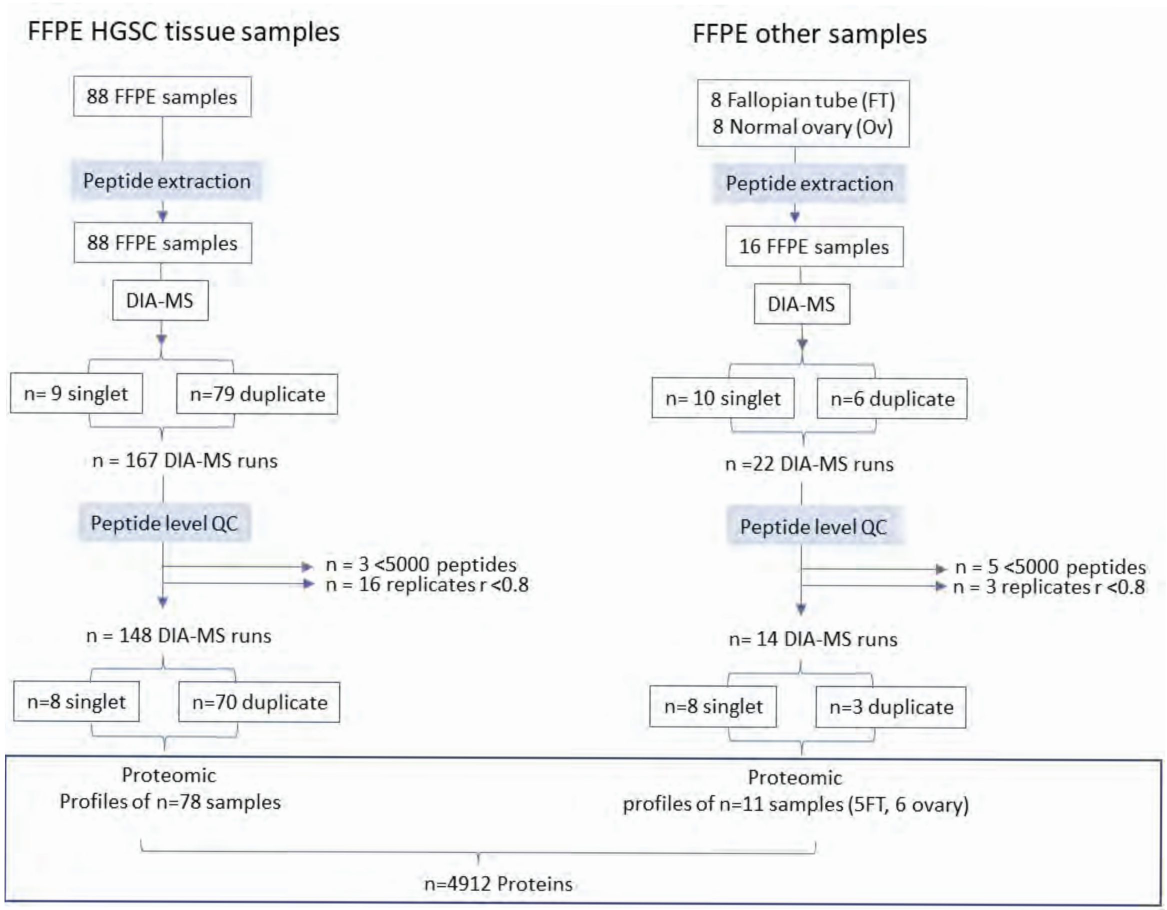

B

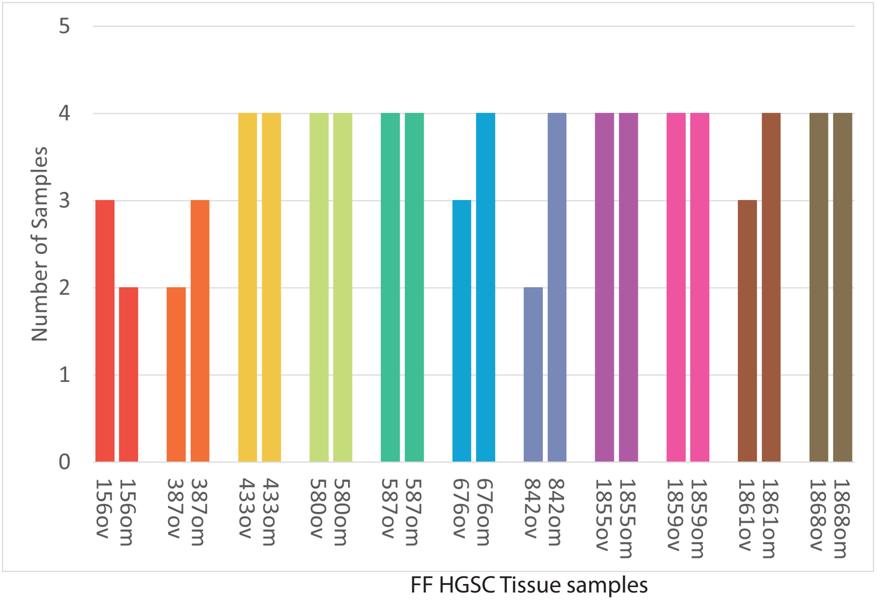

Supplementary Figure 5 – Sampling of FFPE tissue for DIA-MS

A. Process flow of individual FFPE tissue samples from sample collection to the final protein matrix. HEK293 cell line control samples and peptide extracts of selected FF tissue samples were analysed by DIA-MS runs.

B. The final number of individual FFPE HGSC samples contributing data to the analysis (n=78).

Supplementary Material References

1. Newman, A. M. et al. Determining cell type abundance and expression from bulk tissues with digital cytometry. Nat Biotechnol 37, 773-782, doi:10.1038/s41587-019-0114-2 (2019).

2. McDermott, J. E. et al. Proteogenomic Characterization of Ovarian HGSC Implicates Mitotic Kinases, Replication Stress in Observed Chromosomal Instability. Cell Rep Med 1, doi:10.1016/j.xcrm.2020.100004 (2020).

3. Popova, T. et al. Genome Alteration Print (GAP): a tool to visualize and mine complex cancer genomic profiles obtained by SNP arrays. Genome Biol 10, R128, doi:10.1186/gb-2009-10-11-r128 (2009).

Supplementary Tables

Supplementary Table 1. Clinical characteristics of patient cohort

Supplementary Table 2. Pathogenic and likely pathogenic gene variants found in a panel of 30 genes commonly altered in HGSC

Supplementary Table 3. Individual proteins levels per sample in fresh frozen tissue and cell line samples

Supplementary Table 4. Stable Discriminative Proteins based on filtering of the FF protein matrix outlined in Figure 2A; proteins expressed in normal ovary and fallopian tube; proteins in Module 5, dsDNA sensing / inflammation (DSI) score; proteins differentially expressed in samples from HR intact and HR deficient HGSC.

Supplementary Table 5. Genomic lesion scores from whole genome SNP arrays and predicted homologous recombination DNA repair state

Supplementary Table 6. Number of core and core segments and tissue composition of samples included in the analysis

Supplementary Table 7. FFPE tissue sample protein matrix

Supplementary Table 8. Gene level DNA copy number values in units (copy number - 2) so that no amplification or deletion is 0, genes with amplifications have positive values and genes with deletions are negative values (GISTIC 2.0).

Supplementary Table 9. Normalised gene expression values from RNAseq (Transcript per million, TPM)

Supplementary Table 10. Probability scores, entropy values and overall predicted HGSC gene expression PrOTYPE subtype
