# Supplementary material for: Overcoming intra-tumoral heterogeneity for biomarker discovery in the high-grade serous ovarian cancer proteome
Source: NPJ Precis Oncol. 2025 Jun 11;9:172. doi: 10.1038/s41698-025-00911-y (PMC12159146; doi:10.1038/s41698-025-00911-y)
Supplement: Supplementary file 3 — Supplementary AOCS Group Members [file 41698_2025_911_MOESM3_ESM.docx]

**AOCS STUDY GROUP**

***Management Group****:* D Bowtell^1,2^, G Chenevix-Trench^3^, A Green^3^, P Webb^3^, A DeFazio^4,5,6^, D Gertig^7^

***Project and Data Managers****:* N Traficante^1,2^, S Fereday^1,2^, S Moore^3^, J Hung^4^, K Harrap^3^, T Sadkowsky^3^, N Pandeya^3^

***Research Nurses and Assistants***:

L Bowes^1^, L Galletta^1^, D Giles^1^, J Hendley^1^, K Alsop^1,2^, B Alexander^3^, P Ashover^3^, S Brown^3^, T Corrish^3^, L Green^3^, L Jackman^3^, K Ferguson^3^, K Martin^3^, A Martyn^3^, B Ranieri^3^, M Malt^3^, YE Chiew^4^, A Stenlake^6^, H Sullivan^6^, A Mellon^8^, R Robertson^8^, T Vanden Bergh^9^, M Jones^9^, P Mackenzie^9^, J Maidens^10^, K Nattress^11^, J White^12^, V Jayde^13^, P Mamers^14^, T Schmidt^15^, H Shirley^15^, S Viduka^15^, H Tran^15^, S Bilic^15^, L Glavinas^15^, C Ball^16^, C Young^16^, Julia Brooks^17^

***Clinical and Scientific Collaborators****:*

L Mileshkin^1^, G Au-Yeung^1^, K Phillips^1^, D Rischin^1^, N Burdett^1^, R Delahunty^1^, E Christie^1,2^, D Garsed^1,2^, S Fox^1^, D Johnson^1^, S Lade^1^, M Loughrey^1^, N O’Callaghan^1^, W Murray^1^, D Purdie^3^, D Whiteman^3^, A Proietto^8^, S Braye^8^, G Otton^8^, C Camaris^9^, R Crouch^9^, L Edwards^9^, N Hacker^9^, D Marsden^9^, G Robertson^9^, D Bell^10^, S Baron-Hay^10^, A Ferrier^10^ (*dec.*), G Gard^10^, D Nevell^10^, N Pavlakis^10^, S Valmadre^10^, B Young^10^, P Beale^11^, J Beith^11^, J Carter^11^, C Dalrymple^11^, R Houghton^11^, P Russell^11^, M Davy^12^, MK Oehler^12^, C Hall^12^, T Dodd^12^, P Blomfield^13^, D Challis^13^, R McIntosh^13^, A Parker^13^, D Healy^14^, T Jobling^14^, T Manolitsas^14^, J McNealage^14^, P Rogers^14^, B Susil^14^, E Sumithran^14^, I Simpson^14^, N Zeps^15^, I Hammond^16^, Y Leung^16^, A McCartney^16^ (*dec.*), R Stuart-Harris^18^, F Kirsten^19^, J Rutovitz^20^, P Clingan^21^, J Shannon^22^, T Bonaventura^23^, J Stewart^23^, S Begbie^24^, A Glasgow^24^, M Friedlander^25^, M Links^26^, J Grygiel^27^, J Hill^28^, A Brand^5,29^, K Byth^29^, P Harnett^5,29^, G Wain^29^, R Jaworski^30^, R Sharma^5,30^, B Ward^31^, D Papadimos^31^, A Crandon^32^, M Cummings^32^, K Horwood^32^, A Obermair^32^, L Perrin^32^, D Wyld^32^, J Nicklin^32,33^, T Healy^34^, K Pittman^34,^ D Henderson^35^, J Miller^36^, J Pierdes^36^, B Brown^37^, R Rome^37^, D Allen^38^, P Grant^38^, S Hyde^38^, R Laurie^38^, M Robbie^38,^ P Waring^39^, V Billson^40^, J Pyman^40^, D Neesham^40^, M Quinn^40^, C Underhill^41^, R Bell^42^, LF Ng^43^, R Blum^44^, V Ganju^45^, M Buck^46,^ I Haviv^47^

^1^ Peter MacCallum Cancer Centre, Melbourne, Victoria, 3000, Australia.

^2^ Sir Peter MacCallum Cancer Centre Department of Oncology, University of Melbourne, Parkville, Victoria, 3052, Australia

^3^ QIMR Berghofer Medical Research Institute, Brisbane, Queensland, 4006, Australia.

^4^ Centre for Cancer Research, The Westmead Institute for Medical Research, Sydney, New South Wales, 2145, Australia

^5^ The University of Sydney, Sydney, New South Wales, 2006, Australia.

^6^ Department of Gynaecological Oncology, Westmead Hospital, Sydney, New South Wales, 2145, Australia.

^7^ Melbourne School of Population and Global Health, University of Melbourne, Parkville, Victoria, 3052, Australia.

^8^ John Hunter Hospital, Lookout Road, New Lambton, New South Wales, 2305, Australia

^9^ Royal Hospital for Women, Barker Street, Randwick, New South Wales, 2031, Australia

^10^ Royal North Shore Hospital, Reserve Road, St Leonards, New South Wales, 2065, Australia

^11^ Royal Prince Alfred Hospital, Missenden Road, Camperdown, New South Wales, 2050, Australia

^12^ Royal Adelaide Hospital, North Terrace, Adelaide, South Australia, 5000, Australia

^13^ Royal Hobart Hospital, 48 Liverpool St, Hobart, Tasmania, 7000, Australia

^14^ Monash Medical Centre, 246 Clayton Rd, Clayton, Victoria, 3168, Australia

^15^ Western Australian Research Tissue Network (WARTN), St John of God Pathology, 23 Walters Drive, Osborne Park, Western Australia, 6017, Australia

^16^ Women and Infant's Research Foundation, King Edward Memorial Hospital, 374 Bagot Road, Subiaco, Western Australia, 6008, Australia

^17^ St John of God Hospital, 12 Salvado Rd, Subiaco, Western Australia, 6008, Australia

^18^ Canberra Hospital, Yamba Drive, Garran, Australian Capitol Territory, 2605, Australia

^19^ Bankstown Cancer Centre, Bankstown Hospital, 70 Eldridge Road, Bankstown, New South Wales, 2200, Australia

^20^ Northern Haematology & Oncology Group, Integrated Cancer Centre, 185 Fox Valley Road, Wahroonga, New South Wales, 2076, Australia

^21^ Illawarra Shoalhaven Local Health District, Wollongong Hospital, Level 4 Lawson House, Wollongong, New South Wales, 2500, Australia

^22^ Nepean Hospital, Derby Street, Kingswood, New South Wales, 2747, Australia

^23^ Newcastle Mater Misericordiae Hospital, Edith Street, Waratah, New South Wales, 2298, Australia

^24^ Port Macquarie Base Hospital, Wrights Road, Port Macquarie, New South Wales, 2444, Australia

^25^ Prince of Wales Clinical School, University of New South Wales, New South Wales, 2031, Australia

^26^ St George Hospital, Gray Street, Kogarah, New South Wales, 2217, Australia

^27^ St Vincent’s Hospital, 390 Victoria Street, Darlinghurst, New South Wales, 2010, Australia

^28^ Wagga Wagga Base Hospital, Docker St, Wagga Wagga, New South Wales, 2650, Australia

^29^ Crown Princess Mary Cancer Centre, Westmead Hospital, Westmead, Sydney, New South Wales, 2145, Australia.

^30^ Department of Pathology, Westmead Clinical School, Westmead Hospital, The University of Sydney, New South Wales, 2006, Australia

^31^ Mater Misericordiae Hospital, Raymond Terrace, South Brisbane, Queensland, 4101, Australia

^32^ The Royal Brisbane and Women’s Hospital, Butterfield Street, Herston, Queensland, 4006, Australia

^33^ Wesley Hospital, 451 Coronation Drive, Auchenflower, Queensland, 4066, Australia

^34^ Burnside Hospital, 120 Kensington Road, Toorak Gardens, South Australia, 5065, Australia

^35^ Flinders Medical Centre, Flinders Drive, Bedford Park, South Australia, 5042, Australia

^36^ Queen Elizabeth Hospital, 28 Woodville Road, Woodville South, South Australia, 5011, Australia

^37^ Freemasons Hospital, 20 Victoria Parade, East Melbourne, Victoria, 3002, Australia

^38^ Mercy Hospital for Women, 163 Studley Road, Heidelberg, Victoria, 3084, Australia

^39^ Department of Pathology, University of Melbourne, Parkville, Victoria, 3052, Australia.

^40^ The Royal Women’s Hospital, Parkville, Victoria, 3052, Australia

^41^ Border Medical Oncology, Wodonga, Victoria, 3690, Australia

^42^ Andrew Love Cancer Centre, 70 Swanston Street, Geelong, Victoria, 3220, Australia

^43^ Ballarat Base Hospital, Drummond Street North, Ballarat, Victoria, 3350, Australia

^44^ Bendigo Health Care Group, 62 Lucan Street, Bendigo, Victoria, 3550, Australia

^45^ Peninsula Health, 2 Hastings Road, Frankston, Victoria, 3199, Australia

^46^ Mount Hospital, 150 Mounts Bay Road, Perth, Western Australia 6000, Australia

^47^ Faculty of Medicine, Bar-Ilan University, 8 Henrietta Szold St, Safed, Israel
