# Supplementary material for: Overcoming intra-tumoral heterogeneity for biomarker discovery in the high-grade serous ovarian cancer proteome
Source: NPJ Precis Oncol. 2025 Jun 11;9:172. doi: 10.1038/s41698-025-00911-y (PMC12159146; doi:10.1038/s41698-025-00911-y)
Supplement: Supplementary file 4 — Supplementary AOCS Group Members [file 41698_2025_911_MOESM4_ESM.pdf]

AOCS STUDY GROUP

***Management Group**:* D Bowtell[1,2], G Chenevix-Trench[3], A Green[3], P Webb[3], A DeFazio[4,5,6], D Gertig[7]

***Project and Data Managers**:* N Traficante[1,2], S Fereday[1,2], S Moore[3], J Hung[4], K Harrap[3], T Sadkowsky[3], N Pandeya[3]

Research Nurses and Assistants:
L Bowes[1], L Galletta[1], D Giles[1], J Hendley[1], K Alsop[1,2], B Alexander[3], P Ashover[3], S Brown[3], T Corrish[3], L Green[3], L Jackman[3], K Ferguson[3], K Martin[3], A Martyn[3], B Ranieri[3], M Malt[3], YE Chiew[4], A Stenlake[6], H Sullivan[6], A Mellon[8], R Robertson[8], T Vanden Bergh[9], M Jones[9], P Mackenzie[9], J Maidens[10], K Nattress[11], J White[12], V Jayde[13], P Mamers[14], T Schmidt[15], H Shirley[15], S Viduka[15], H Tran[15], S Bilic[15], L Glavinas[15], C Ball[16], C Young[16], Julia Brooks[17]

Clinical and Scientific Collaborators:
L Mileshkin[1], G Au-Yeung[1], K Phillips[1], D Rischin[1], N Burdett[1], R Delahunty[1], E Christie[1,2], D Garsed[1,2], S Fox[1], D Johnson[1], S Lade[1], M Loughrey[1], N O'Callaghan[1], W Murray[1], D Purdie[3], D Whiteman[3], A Proietto[8], S Braye[8], G Otton[8], C Camaris[9], R Crouch[9], L Edwards[9], N Hacker[9], D Marsden[9], G Robertson[9], D Bell[10], S Baron-Hay[10], A Ferrier[10] (*dec.*), G Gard[10], D Nevell[10], N Pavlakis[10], S Valmadre[10], B Young[10], P Beale[11], J Beith[11], J Carter[11], C Dalrymple[11], R Houghton[11], P Russell[11], M Davy[12], MK Oehler[12], C Hall[12], T Dodd[12], P Blomfield[13], D Challis[13], R McIntosh[13], A Parker[13], D Healy[14], T Jobling[14], T Manolitsas[14], J McNealage[14], P Rogers[14], B Susil[14], E Sumithran[14], I Simpson[14], N Zeps[15], I Hammond[16], Y Leung[16], A McCartney[16] (*dec.*), R Stuart-Harris[18], F Kirsten[19], J Rutovitz[20], P Clingan[21], J Shannon[22], T Bonaventura[23], J Stewart[23], S Begbie[24], A Glasgow[24], M Friedlander[25], M Links[26], J Grygiel[27], J Hill[28], A Brand[5,29], K Byth[29], P Harnett[5,29], G Wain[29], R Jaworski[30], R Sharma[5,30], B Ward[31], D Papadimos[31], A Crandon[32], M Cummings[32], K Horwood[32], A Obermair[32], L Perrin[32], D Wyld[32], J Nicklin[32,33], T Healy[34], K Pittman[34,] D Henderson[35], J Miller[36], J Pierdes[36], B Brown[37], R Rome[37], D Allen[38], P Grant[38], S Hyde[38], R Laurie[38], M Robbie[38,] P Waring[39], V Billson[40], J Pyman[40], D Neesham[40], M Quinn[40], C Underhill[41], R Bell[42], LF Ng[43], R Blum[44], V Ganju[45], M Buck[46,] I Haviv[47]

[1] Peter MacCallum Cancer Centre, Melbourne, Victoria, 3000, Australia.

[2] Sir Peter MacCallum Cancer Centre Department of Oncology, University of Melbourne, Parkville, Victoria, 3052, Australia

[3] QIMR Berghofer Medical Research Institute, Brisbane, Queensland, 4006, Australia.

[4] Centre for Cancer Research, The Westmead Institute for Medical Research, Sydney, New South Wales, 2145, Australia

[5] The University of Sydney, Sydney, New South Wales, 2006, Australia.

[6] Department of Gynaecological Oncology, Westmead Hospital, Sydney, New South Wales, 2145, Australia.

[7] Melbourne School of Population and Global Health, University of Melbourne, Parkville, Victoria, 3052, Australia.

[8] John Hunter Hospital, Lookout Road, New Lambton, New South Wales, 2305, Australia
[9] Royal Hospital for Women, Barker Street, Randwick, New South Wales, 2031, Australia
[10] Royal North Shore Hospital, Reserve Road, St Leonards, New South Wales, 2065, Australia
[11] Royal Prince Alfred Hospital, Missenden Road, Camperdown, New South Wales, 2050, Australia
[12] Royal Adelaide Hospital, North Terrace, Adelaide, South Australia, 5000, Australia
[13] Royal Hobart Hospital, 48 Liverpool St, Hobart, Tasmania, 7000, Australia
[14] Monash Medical Centre, 246 Clayton Rd, Clayton, Victoria, 3168, Australia
[15] Western Australian Research Tissue Network (WARTN), St John of God Pathology, 23 Walters Drive, Osborne Park, Western Australia, 6017, Australia
[16] Women and Infant's Research Foundation, King Edward Memorial Hospital, 374 Bagot Road, Subiaco, Western Australia, 6008, Australia
[17] St John of God Hospital, 12 Salvado Rd, Subiaco, Western Australia, 6008, Australia
[18] Canberra Hospital, Yamba Drive, Garran, Australian Capitol Territory, 2605, Australia
[19] Bankstown Cancer Centre, Bankstown Hospital, 70 Eldridge Road, Bankstown, New South Wales, 2200, Australia
[20] Northern Haematology & Oncology Group, Integrated Cancer Centre, 185 Fox Valley Road, Wahroonga, New South Wales, 2076, Australia
[21] Illawarra Shoalhaven Local Health District, Wollongong Hospital, Level 4 Lawson House, Wollongong, New South Wales, 2500, Australia
[22] Nepean Hospital, Derby Street, Kingswood, New South Wales, 2747, Australia
[23] Newcastle Mater Misericordiae Hospital, Edith Street, Waratah, New South Wales, 2298, Australia
[24] Port Macquarie Base Hospital, Wrights Road, Port Macquarie, New South Wales, 2444, Australia
[25] Prince of Wales Clinical School, University of New South Wales, New South Wales, 2031, Australia
[26] St George Hospital, Gray Street, Kogarah, New South Wales, 2217, Australia
[27] St Vincent's Hospital, 390 Victoria Street, Darlinghurst, New South Wales, 2010, Australia
[28] Wagga Wagga Base Hospital, Docker St, Wagga Wagga, New South Wales, 2650, Australia
[29] Crown Princess Mary Cancer Centre, Westmead Hospital, Westmead, Sydney, New South Wales, 2145, Australia.
[30] Department of Pathology, Westmead Clinical School, Westmead Hospital, The University of Sydney, New South Wales, 2006, Australia
[31] Mater Misericordiae Hospital, Raymond Terrace, South Brisbane, Queensland, 4101, Australia
[32] The Royal Brisbane and Women's Hospital, Butterfield Street, Herston, Queensland, 4006, Australia

[33] Wesley Hospital, 451 Coronation Drive, Auchenflower, Queensland, 4066, Australia
[34] Burnside Hospital, 120 Kensington Road, Toorak Gardens, South Australia, 5065, Australia
[35] Flinders Medical Centre, Flinders Drive, Bedford Park, South Australia, 5042, Australia
[36] Queen Elizabeth Hospital, 28 Woodville Road, Woodville South, South Australia, 5011, Australia
[37] Freemasons Hospital, 20 Victoria Parade, East Melbourne, Victoria, 3002, Australia
[38] Mercy Hospital for Women, 163 Studley Road, Heidelberg, Victoria, 3084, Australia
[39] Department of Pathology, University of Melbourne, Parkville, Victoria, 3052, Australia.
[40] The Royal Women's Hospital, Parkville, Victoria, 3052, Australia
[41] Border Medical Oncology, Wodonga, Victoria, 3690, Australia
[42] Andrew Love Cancer Centre, 70 Swanston Street, Geelong, Victoria, 3220, Australia
[43] Ballarat Base Hospital, Drummond Street North, Ballarat, Victoria, 3350, Australia
[44] Bendigo Health Care Group, 62 Lucan Street, Bendigo, Victoria, 3550, Australia
[45] Peninsula Health, 2 Hastings Road, Frankston, Victoria, 3199, Australia
[46] Mount Hospital, 150 Mounts Bay Road, Perth, Western Australia 6000, Australia
[47] Faculty of Medicine, Bar-Ilan University, 8 Henrietta Szold St, Safed, Israel
